# Adapting the Stress First Aid Model for Frontline Healthcare Workers during COVID-19

**DOI:** 10.3390/ijerph21020171

**Published:** 2024-02-01

**Authors:** Mayer H. Bellehsen, Haley M. Cook, Pooja Shaam, Daniella Burns, Peter D’Amico, Arielle Goldberg, Mary Beth McManus, Manish Sapra, Lily Thomas, Annmarie Wacha-Montes, George Zenzerovich, Patricia Watson, Richard J. Westphal, Rebecca M. Schwartz

**Affiliations:** 1 Center for Traumatic Stress, Resilience and Recovery at Northwell Health, Great Neck, NY 11021, USA; mbellehsen@northwell.edu (M.H.B.); pshaam@northwell.edu (P.S.); dburns1@northwell.edu (D.B.); agoldberg9@northwell.edu (A.G.); awachamontes@northwell.edu (A.W.-M.); gzenzerovich@northwell.edu (G.Z.); rschwartz3@northwell.edu (R.M.S.); 2 Department of Psychiatry, Zucker Hillside Hospital at Northwell Health, Glen Oaks, NY 11004, USA; pdamico@northwell.edu (P.D.); msapra@northwell.edu (M.S.); 3 Behavioral Health Service Line, Northwell Health, New York, NY 10022, USA; mmcmanus@northwell.edu; 4 Department of Occupational Medicine, Epidemiology and Prevention, Donald and Barbara Zucker School of Medicine at Hofstra/Northwell, Great Neck, NY 11021, USA; 5 Institute for Nursing, Northwell Health, New Hyde Park, NY 11042, USA; lthomas@northwell.edu; 6 National Center for PTSD, White River Junction, VT 05009, USA; patricia.j.watson@dartmouth.edu; 7 School of Nursing, University of Virginia, Charlottesville, VA 22903, USA; richard.westphal@virginia.edu

**Keywords:** Stress First Aid, nurses, COVID-19, resilience, burnout, stress

## Abstract

The coronavirus pandemic has generated and continues to create unprecedented demands on our healthcare systems. Healthcare workers (HCWs) face physical and psychological stresses caring for critically ill patients, including experiencing anxiety, depression, and posttraumatic stress symptoms. Nurses and nursing staff disproportionately experienced COVID-19-related psychological distress due to their vital role in infection mitigation and direct patient care. Therefore, there is a critical need to understand the short- and long-term impact of COVID-19 stress exposures on nursing staff wellbeing and to assess the impact of wellbeing programs aimed at supporting HCWs. To that end, the current study aims to evaluate an evidence-informed peer support stress reduction model, Stress First Aid (SFA), implemented across units within a psychiatric hospital in the New York City area during the pandemic. To examine the effectiveness of SFA, we measured stress, burnout, coping self-efficacy, resilience, and workplace support through self-report surveys completed by nurses and nursing staff over twelve months. The implementation of SFA across units has the potential to provide the workplace-level and individual-level skills necessary to reduce stress and promote resilience, which can be utilized and applied during waves of respiratory illness acuity or any other healthcare-related stressors among this population.

## 1. Introduction

The COVID-19 pandemic placed an unprecedented burden on healthcare workers (HCWs). It also highlighted pre-existing stressors that exacerbate challenges within hospital systems across the country [1,2]. HCWs experience high levels of stress due to the nature of their job responsibilities, including caring for critically ill and injured patients, patient deaths, increased workloads, and long hours [3]. These occupational stressors are often exacerbated during periods of higher healthcare demand, such as during a disaster or public health emergency [4,5]. Occupationally, HCWs are particularly vulnerable to workplace stressors, conferring increased risk for posttraumatic stress reactions [5,6]. Indeed, an analysis of the 2008 American Community Survey and National Death Index records through 2019 found that healthcare workers were at increased risk of suicide compared with non-healthcare workers, specifically registered nurses, healthcare support workers, and health technicians [7]. National and international studies of HCWs’ occupational and health outcomes during the COVID-19 pandemic have shown increased rates of psychopathology including anxiety, depression, and posttraumatic stress; behavioral changes including sleep disturbance, relationship difficulties, and substance use; and increased rates of burnout, compassion fatigue, and job dissatisfaction [1,2,6,8,9,10].

Approximately two years after the first wave of the pandemic, the US Bureau of Labor Statistics estimated that there were over three million employed registered nurses, the largest healthcare occupation in the US, of which nearly 88% identify as female [10,11]. Nursing staff report increased rates of moral injury stemming from treatment decisions based on limited resources, increases in stress from the volume and intensity of their work, and a new sense of vulnerability and fear for their safety and the safety of their loved ones [12,13]. Similar outcomes were found among nurses across triage and non-triage hospitals in Egypt during the pandemic, wherein occupational stressors including high workload, exposure to death, personal fears, and stigma were associated with higher stress, decreased job satisfaction, and increased intent to leave their current position [14]. In a longitudinal study of Italian healthcare workers between May 2020 and July 2021, the authors found subclinical levels of psychiatric symptoms, including stress, depression, state anger, and emotional exhaustion, across the three time points measured in the study, hypothesized to indicate a higher baseline level of resilience among HCWs [15]. Related measures of emotional and occupational wellbeing, however, were found to be negatively impacted in a another sample of Italian healthcare workers, during (July 2021) and post (July 2023) pandemic [16]. Specifically, while rates of job burnout and symptoms of depression remained high between time points, rates of compassion fatigue increased following the pandemic [16]. Findings may be suggestive of negative ongoing impacts to emotional and occupational wellbeing experienced by healthcare workers. The collective impact of trauma from COVID-19, as well as subsequent responses to the pandemic at the provider level, contribute to the emotional toll experienced in the wake of COVID-19 on healthcare providers [1,2]. With an understanding of the unique risks experienced by HCWs generally and those experienced explicitly by nurses during the COVID-19 pandemic, targeted interventions are needed to address provider wellbeing.

### 1.1. Peer Support Intervention

The relationship between occupational stress and adverse psychological outcomes is well established [17,18,19,20]. Peer support programs have been cited as potentially effective for addressing HCW wellbeing in the context of COVID-19 [21]. Generally, positive relationships with supervisors and coworkers have been associated with lower workplace stress [22]. Social support is recognized as a leading driver for this positive association [23]. Specifically, in Karasek’s 1982 job strain model, coworker and supervisor support is understood to moderate associations between general occupational stressors, job control, and mental strain [24]. In a further examination of the role of supervisor and coworker support, after controlling for negative affect, high levels of supervisor support reduced the negative effects of job strain on level of job satisfaction, while coworker support mitigated the association between job strain and work performance [25]. Peer support models aid in the deconstruction of mental health stigma through increasing awareness and verbal expression of stress individually and as a team [23]. This is particularly relevant for HCWs whose occupation requires attention to and caring for others, often at their own expense [13]. Studies indicated that HCWs grappling with stressors from COVID-19 are interested in obtaining support, but few employees currently utilize emotional support resources [24].

The expansion of peer support services to address HCW needs during the COVID-19 pandemic represented an essential shift in occupational health [25,26,27]. In response to the pandemic, several hospitals and health systems have implemented hotlines and brief individual- or group-based supportive telehealth sessions for employees [28,29]. Support services have focused on the screening for and provision of mental health services at the individual, small group, and department levels, providing in-house stress-inoculation and resilience-promoting training, and, when clinically indicated, connecting HCWs to external referrals for ongoing care [25,26,27]. Additional services have included the dissemination of resources through online platforms (i.e., websites, apps), wherein employees can access online resources to support mental and physical health, psychosocial stressors (e.g., parenting and caregiving during COVID-19), and resilience maintenance. Initial qualitative findings from a hospital-based peer support program found shifts in organizational culture, staff skill building for recognizing and supporting coworkers in distress, and support for individuals already providing psychosocial peer support [30]. While the introduction and utilization of these services is a crucial step, the need for comprehensive, evidence-informed interventions remains.

A growing understanding of the negative impacts of COVID-19 on HCWs highlights the need for the development and evaluation of comprehensive, effective, and sustainable interventions to address the acute and long-term health needs of healthcare providers. To date, research exploring the use of empirically supported, individual-level treatments (e.g., cognitive behavioral therapy (CBT)) to improve mental health and wellbeing during the pandemic stop short of addressing resilience processes and contextual factors that impact wellbeing, such as perceptions of workplace support [17,26,28,29,31]. A systematic review published in the Cochrane Database determined there is a paucity of evidenced-based effectiveness research on interventions aimed at supporting the resilience and mental health of HCWs during and after disasters, asserting a strong need for studies examining comprehensive interventions for HCWs during COVID-19 [32]. The Systems Model theoretical framework delineates the various workplace levels that are impacted by stress and burnout, such as unit/team, leadership, and the healthcare industry [33]. For instance, at the workplace level, the Systems Model posits that stress and burnout will also be associated with negative consequences such as increased absenteeism, turnover, and lower engagement. The Systems Model also highlights individual and work system factors that might mediate stress and burnout, including coping strategies and coping self-efficacy, resilience, and organizational social support.

### 1.2. Stress First Aid

SFA is a peer support and self-care model involving the promotion of social support and resilience-building to mitigate stress reactions, individually and within teams [34,35,36,37]. Initially developed in the context of high-risk occupations (i.e., military service, fire and rescue, law enforcement), SFA has shown positive preliminary results for improving perceived ability to respond to behavioral health issues among teams [38]. SFA has, more recently, been adapted to meet the needs of HCWs. Specifically, SFA for HCWs is designed to promote peer-support interventions that have the potential to impact SFA self-efficacy, or the ability to recognize stress reactions in oneself and colleagues, encourage increased awareness and utilization of occupational wellbeing resources, improve perceptions of workplace support, and shift workplace stress and burnout levels. Stress First Aid is inherently compatible with Karasek’s model; when demand is high, as was seen among HCWs during the pandemic, SFA seeks to increase control and social support within the workplace by enabling more effective communication around stressors and procurement of support, with the intent to decrease the negative impacts of job strain, including burnout. Stress First Aid acts as an effective mechanism for aligning efforts between programs as well with the goal of facilitating knowledge and awareness of employee supports such as EAP. This connection and cooperation promote resource-sharing and aid in the implementation of informed and efficient efforts in supporting our employees and the community.

**Figure 1:**
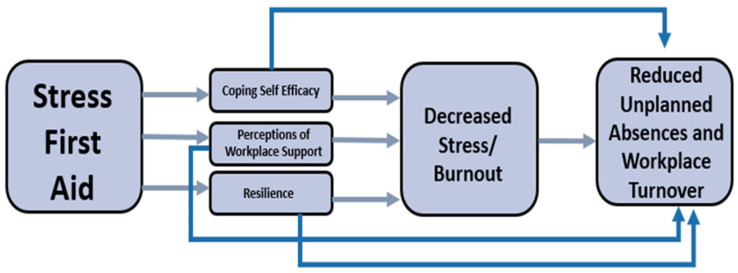
Impact of Stress First Aid.

SFA utilizes empirically informed elements that guide efforts for offering peer support actions in response to ongoing adversity, trauma, or disasters [34,39,40,41]. The five empirically informed elements include the promotion of a sense of (1) safety, (2) calm, (3) connectedness, (4) self- and community-efficacy, and (5) hope [42]. These directly map onto five of the seven Core Actions (7 C’s) of SFA: cover, calm, connect, competence, and confidence. Check and coordinate are added as continuous core actions for situational monitoring of stress and linkage into care as needed. SFA’s seven core actions directly impact coping self-efficacy, resilience, and perceptions of organizational support. Self-efficacy in terms of one’s ability to cope with stress, has been linked with psychological stress itself as well as stress-related burnout, mental health symptoms, and workplace turnover intentions [43,44,45,46]. SFA is designed to generate increased individual and team coping and self-efficacy skills, in addition to improvements in leadership, workplace resilience, improved knowledge of and use of resources, and organizational social support. The SFA framework posits that stress injuries often result in decreased self-awareness of stress. This gap widens further for HCWs, who, by nature of the occupation, employ ‘other’ rather than ‘self’ focus and, therefore, are more likely to provide peer support than self-care. Thus, the provision of services within SFA includes direct peer support, wherein team members learn how to identify, communicate, and respond to stress. Initial research findings support the feasibility and acceptability of SFA among firefighter and nursing populations [40]. Figure 1 provides an overview of the anticipated impact of SFA.

The ability to test the impacts of SFA on the aforementioned constructs in the healthcare setting is important as there is limited research on its impact in the healthcare setting. SFA was developed with input from HCWs so that it would be feasible and acceptable for use with this population. SFA’s flexible structure easily allows for these types of adaptations. For example, the lives of HCWs, particularly during a pandemic, are hectic and intensely busy. It was imperative that learning the basic framework of SFA would be a quick process. Healthcare settings can implement Stress First Aid setting-wide with 15 min briefings to raise awareness and educate about the importance of using SFA principles for stress mitigation. SFA champions or coaches can then train, mentor, and provide ongoing resources in their units. They also regularly assess the unit’s overall stress levels on the stress continuum and shape the dialogue about the resources that exist to build resilience and capacity. This type of implementation is consistent with the EU Field Guide to Managing Complexity (and Chaos) in times of crisis, which recommends using a flexible approach built upon research- or theory-informed frameworks that empower adaptive actions in the context of complex public health situations [47]. It is also consistent with qualitative findings from a Cochrane review regarding work-related resilience interventions in the context of disease outbreaks [34,47]. This review noted that the successful implementation of programs depends upon flexible interventions that are culturally appropriate and adaptable to local needs. Also important are effective communication, cohesion through networks, a positive learning climate where team members feel valued and a part of the change process, and sufficient time and space for reflective thinking and evaluation. When HCWs are overwhelmed, they appreciate a “map” that can guide them through a variety of circumstances at whatever level they have the capacity for. SFA also allows for a long-term approach that includes multiple strategies for self-care strategies based on stress levels and current concerns, as well as sharing and learning from colleagues, consulting with mentors, and making time for the small actions that provide support.

### 1.3. Present Study

This study aims to address a gap in the current literature regarding the implementation and efficacy of a comprehensive, evidence-based peer support program in a cohort of HCWs at a large psychiatric hospital during the COVID-19 pandemic. The current study uses a longitudinal design to best capture fluctuations in outcomes over time and to understand both the short and longer-term potential impacts of SFA. The authors hypothesize that, over 12 months, SFA will be highly utilized, well-received, and efficacious in increasing participants’ (a) self-efficacy, (b) resilience, (c) awareness and utilization of wellbeing resources, and (d) perceptions of organizational support. Secondary/distal outcomes will also be explored, including SFA’s impact on stress and burnout.

## 2. Materials and Methods

### 2.1. Participants

To evaluate the effectiveness, implementation, and maintenance of the SFA program, 562 nurses and nursing support staff in a psychiatric hospital in the metropolitan New York area were given measures that were collected at baseline (before implementation), three, six, nine, and twelve months post SFA implementation to examine how each of the proximal and distal outcomes changes over the five-time points. Convenience sampling approaches were utilized to send each of the identified 562 HCWs the electronic survey. All data were collected and stored in a Research Electronic Data Capture (REDCap) database, which is a HIPAA-compliant database that requires a password and only allows access to specific individuals added by the CTSRR team. Data were collected between May 2021 and June 2022 via a REDCap electronic health questionnaire that was directly emailed to eligible participants. All study measurements, including SFA self-efficacy, wellbeing resource awareness/utilization, perceptions of organizational support, resilience, stress, and burnout, were contained within this questionnaire. Nurses and nursing staff who were working in an inpatient capacity at the hospital between May 2021 and June 2022 were included. Of the 562 nurses and nursing staff that were sent the survey, 150 participants completed the survey at baseline, 128 at the 3-month follow-up, 111 at the 6-month follow-up, 92 at the 9-month follow-up, and 116 at the 12-month follow-up. Table 1 shows the role breakdown of the 266 participants who completed the survey at any time point. Of the total sample, 77% consisted of mental health workers and registered nurses, with survey participants across 14 units of a single psychiatric hospital.

### 2.2. SFA Procedure

Implementation of SFA into a large hospital system entailed a four-step process that included preparation of teams, training, integration, and sustainment. SFA program staff included designated SFA Coaches expertly trained in SFA by two developers of SFA and project management support. The SFA program staff prepared a particular hospital team by orienting them to a process for implementation. Hospitals would identify an SFA site coordination and other personnel to support operations and report to executive sponsors. The SFA program team would help the hospital coordination team identify personnel that could facilitate training and team members that would support integration. Additional awareness raising activities such as briefings, newsletter announcements, and kickoff events were conducted during the preparation phase. SFA Coaches also prepared two levels of training- one for leaders and one for remaining staff. The coaches then provided master training for site trainers to deliver these trainings in more intimate settings to their staff and the coaches met regularly with the trainers to monitor them and provide feedback. SFA Coaches were also available to support responses to critical incidents. Critical to the SFA roll out was an integration process to embed SFA into daily team operations to moderate ongoing and exceptional occupational stressors across hospital units. Once about 50% of a site was trained, a 12-week integration phase was initiated in which leaders were asked to begin practices of “Raising Awareness”, “Growing the Green”, and “Stopping the Burn”. This corresponded onto actions of posting signage, actively engaging in SFA practices (like a color check in and calming activities), and responding to elevated stress levels, respectively. The process of integration was monitored with self-report surveys through REDCap and a process of auditing. Lastly, sustainment was achieved by embedding check-ins on progress with SFA into hospital-wide administrative meetings, embedding practices like color checks into daily safety checks and weekly rounding, and by embedding training into new hire orientation so that new staff were trained. Progress with each step of the process was monitored with various process metrics.

### 2.3. Measures

We utilized a secure, HIPAA-compliant database, REDCap, for all data collection. The Human Resources department provided the contact information of all eligible nurses and nursing staff to the CTSRR team, which was then used to create a unique ID for each participant within REDCap. All study measurements were contained within the electronic baseline questionnaire, which was emailed directly to eligible participants through a secure and unique REDCap link at each time point. Responses are linked to a participant’s record, where they can be tracked and analyzed.

#### 2.3.1. Proximal Outcomes

Proximal outcomes include resilience, SFA self-efficacy, and perceptions of workplace support at each time point. The Connor–Davidson Resilience Scale 2© (CD-RISC 2) is a two-item scale that is useful as a brief measure of resilience to assess one’s ability to adapt to change and bounce back after hardship [48]. The two items include “I am able to adapt when changes occur” and “I tend to bounce back after illness, injury, or other hardships”. Each item on the CD-RISC 2 has responses that range between 1–5 (*1 = not true at all*, *2 = rarely true*, *3 = sometimes true*, *4 = often true*, *5 = true nearly all the time*). Item scores are summed for a total score range of 2–10 with higher scores on this screener indicative of greater levels of resilience.

The SFA self-efficacy score was developed by the research team to better understand SFA implementation (confidence in addressing the seven core actions of SFA), including confidence in one’s ability to identify stress in co-workers, take action to reduce stress in co-workers, and link stressed co-workers into care. Items include “How confident are you in your ability to identify the level of risk/distress of an impacted person (i.e., self or co-worker) during and following a stressful event?”; “How confident are you in your knowledge of resources to link a stress-impacted person to needed supports?”; and “How confident are you in your ability to take action to reduce the stress of an affected person/s (i.e., calm, cover, connect, competence, confidence)?”. Item responses ranged from 1–4 (*1 = not confident*, *2 = a little confident*, *3 = confident*, *4 = very confident*). Scores were summed for a total range of 3–12 with higher scores hypothesized to indicate greater SFA self-efficacy.

Perceived organizational support was measured using the Deployment Risk and Resilience Inventory-2 (DRRI-2), unit support subscale, adapted for use with frontline HCWs, which is a 12-item instrument with each item response between 1 and 5 (1 = strongly disagree; 5 = strongly agree) [45]. Total DRRI-2, Unit Support subscale score is created by summing each item rating with higher scores indicative of greater perceived social support from co-workers/unit members/unit leaders (range: 12–60). We aimed to measure co-worker and manager interest in employee wellbeing. Participants were asked whether “co-workers on my unit are interested in my wellbeing” and “the leaders of my unit are interested in my personal welfare”, with responses between 1 and 5 (*1 = strongly disagree*, *2 = disagree*, *3 = neither agree nor disagree*, *4 = agree*, *5 = strongly agree*).

#### 2.3.2. Distal Outcomes

Distal outcomes included stress and burnout levels. The Stress Continuum Model is a foundational part of the SFA model that helps with the assessment of stress response levels, ranging from *Green* (exceptionally low stress and stress reactions) through *Yellow* and *Orange* to *Red* (significant exposure to stress and experience of stress reactions) categories. Participants were asked to rate their level of stress for the past two weeks on a scale of 1–4, with *1* being the least stressful and *4* being the most stressful, and with the following corresponding colors: 1—green; 2—yellow; 3—orange; 4—red.

The Adapted Maslach Burnout Inventory (MBI) measured nine emotional exhaustion (EE) items, for example, “I feel burned out from my work”, and five depersonalization (DP) items, including “I’ve noticed that I’ve become more callous (i.e., less sympathetic/compassionate) toward patients/customers”. Burnout domains were evaluated on a scale of 1–5 (*1 = strongly disagree*, *2 = disagree*, *3 = neither agree nor disagree*, *4 = agree*, *5 = strongly agree*). For both subscales, higher mean scores are indicative of higher degrees of burnout.

To better understand the utilization of wellbeing resources, participants were asked to endorse a list of fourteen hospital-provided wellbeing resources that they are aware of and have utilized or directed a coworker to in the last three months. Examples of responses include the *Employee & Family Assistance Program (EAP)*, *Team Lavender*, *Chaplain on-site support*, and the *Emotional Support Resource Call Center.*

Lastly, demographic data were collected including, position/job role, primary worksite/hospital, and primary work unit/department.

### 2.4. Statistical Analyses

All longitudinal analyses were conducted using the Generalized Estimating Equations (GEE) approach [49] under the assumption that the missing values were missing completely at random (MCAR). The working correlation structures considered for fitting GEE models for each variable were ‘Independent’, ‘Exchangeable’, ‘AR-1’, ‘3-Dependent’, and ‘Unstructured’. The criteria used for selecting the best covariance structure for each variable included both the Quasi Likelihood Information Criterion (QIC) values as well as a comparison between the model-based estimates and the empirical estimates of the correlation matrix [50]. Empirical estimators were obtained using the sandwich estimator [50]. PROC GENMOD in SAS version 9.4 was used for all GEE analyses. Marginal means for the continuous dependent variables and the odds for the binary dependent variables across all time points were estimated using the ‘least squares means’ option within the GENMOD procedure.

## 3. Results

### 3.1. Longitudinal Descriptive of Study Variables

Table 2 provides descriptive characteristics for participants’ levels of self-efficacy, resilience, perceptions of organizational support, stress, burnout, and awareness of resources from baseline through 12-month follow-up.

### 3.2. Generalized Estimating Equation (GEE) Analysis of Study Variables

**Figure 2:**
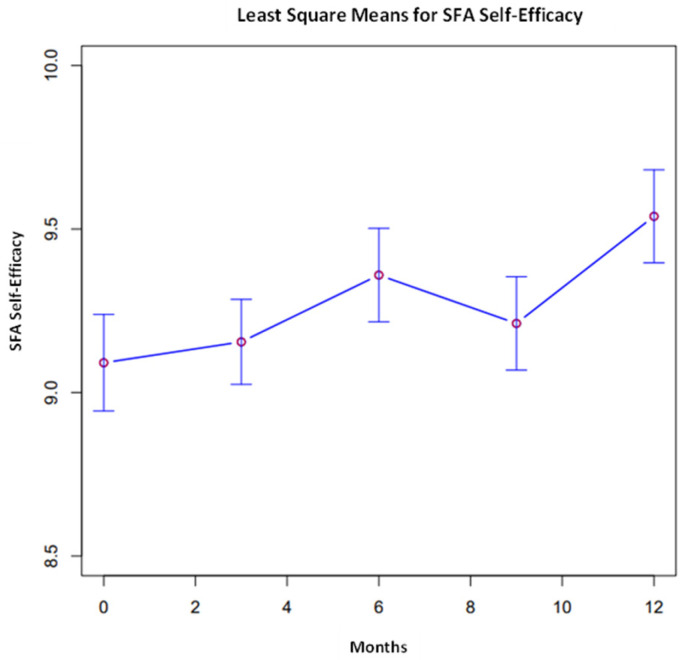
SFA self-efficacy across all time points.

**Figure 3:**
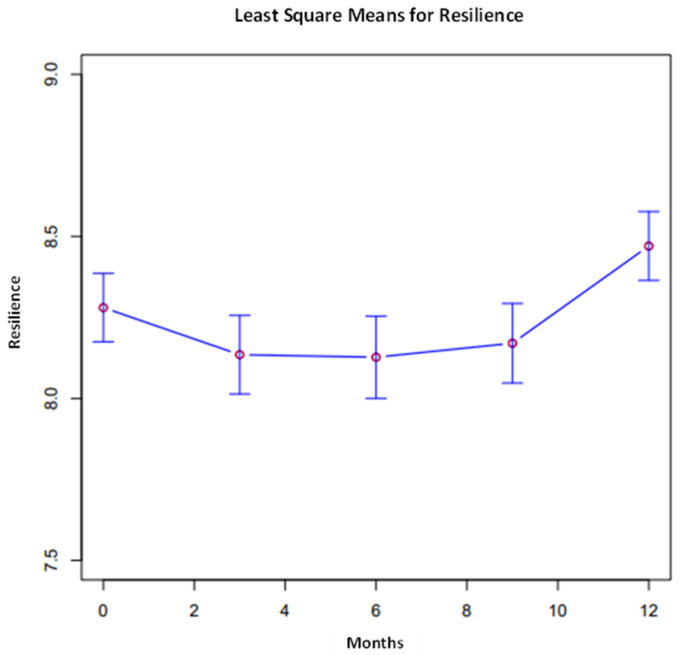
Resilience across all time points.

**Figure 4:**
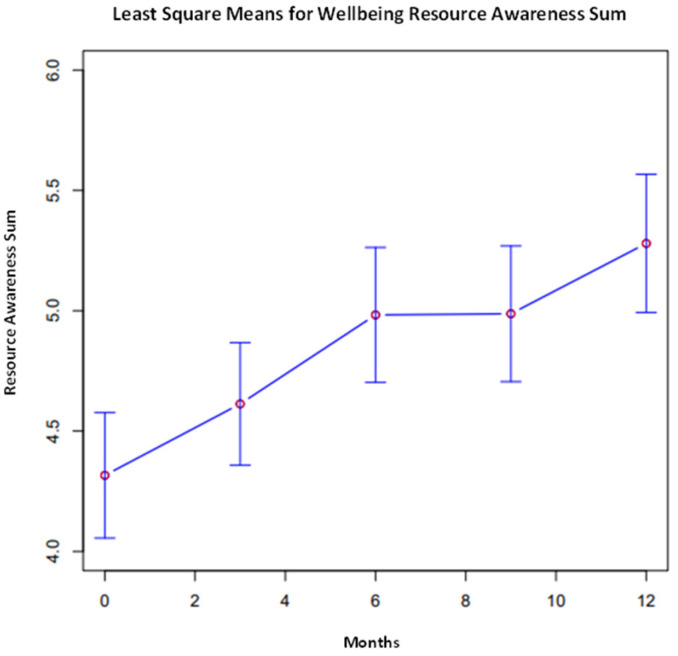
Awareness of wellbeing resources across all time points.

The overall comparisons of least square means were conducted across time points for each variable, presented in Figure 1, Figure 2 and Figure 3. Proximal outcomes of and SFA self-efficacy ((χ^2^, 4) = 9.45, *p* = 0.051) and resilience ((χ^2^, 4) = 11.47, *p* < 0.05) significantly differed across time points. In addition, the average number of resources participants are aware of significantly differed across timepoints ((χ^2^, 4) = 9.55, *p* < 0.05). All outcomes generally increased over time (see Figure 2, Figure 3 and Figure 4).

Mean differences across time points did not significantly differ for the perception of organizational support ((χ^2^, 4) = 5.67, *p* = 0.225), stress ((χ^2^, 4) = 2.44, *p* = 0.656), burnout ((χ^2^, 4) = 6.03, *p* = 0.197), or utilization of resources ((χ^2^, 4) = 2.57, *p* = 0.632). When examined for change between the two-time points of baseline and 12 months, there are two additional significant results. Perceptions of organizational support significantly increased from baseline to 12 months (Mean difference = 0.396; *p* = 0.0278), and there was a borderline significant decrease in burnout (Mean difference = −0.287; *p* = 0.057).

## 4. Discussion

Workplace stress, emphasized during the COVID-19 pandemic, remains a prominent issue among healthcare professionals. The authors of the present study sought to examine the use of Stress First Aid (SFA) as a targeted, self-care, and peer-support intervention to address healthcare worker wellbeing. Proximal outcomes were hypothesized to increase from baseline to 12-month follow-up, including self-efficacy, resource awareness and utilization, resilience, and perceptions of organizational support. At the same time, potential decreases in distal outcomes of stress and burnout were also explored. Generalized estimating equations (GEE) highlighted significant increases across three-month intervals from baseline to 12-month follow-up for self-efficacy, resource awareness, and resilience rates. Additionally, when considering only changes from baseline to 12-month follow-up, a significant increase was found in perceptions of organizational support and a borderline significant decrease in rates of burnout. In the present study, significance was not found for the utilization of resources or rates of stress.

This longitudinal study reveals unique shifts in the data across the 12 months. Rates of SFA self-efficacy increased, then decreased at month nine, followed by an increase at the twelve-month follow-up. A similar pattern emerged for rates of burnout, where, though not significant, participants reported higher rates at month nine before dropping in month twelve. Survey participation was also notably lower at month nine; however, response rates increased again at the twelve-month follow-up. There was a significant spike in COVID-19 cases in January 2022 [51], which may be reflected in the relative decrease in participation and feelings of self-efficacy at month nine, according to data that were collected in February and March 2022. Natural fluctuations in external stressors may account for some of the observed variability; however, a time-matched control group would be needed to further assess this hypothesis.

Another a point of interest is that the rates of resilience stayed fairly consistent during months 3–9, with a marked increase at the 12-month follow-up. Given the extended period of high-acuity work at the height of the pandemic for this sample of nurses, it is feasible that there was less capacity for resilience building initially, when the focus was required to be on addressing basic needs (i.e., fear of contamination) [52]. Resilience, as a construct, is generally measured as a state characteristic, not a trait characteristic, and may require more time under intervention exposure to see a quantifiable shift [53]. A larger sample size and dose/response analysis may be beneficial in determining this threshold for change. It is also likely that the threshold is variable-dependent, with SFA self-efficacy showing its greatest increase at the six-month time point.

### 4.1. Theoretical Implications

SFA aims to build protective factors to increase resilience and mitigate stress reactions. These factors can hopefully contribute longitudinally to decreased distress and burnout, and in this study, some significant change was found between baseline and 12-month follow-up for distal outcomes. However, the authors generally expected to see less change in measures of stress and burnout in the context of implementing SFA during a pandemic. Because significant change was found for distal outcomes even in this context, we are hopeful that in less stressful circumstances, the use of SFA can further contribute to a reduction in stress and burnout, mainly when the organization uses the model to build leadership’s capacity to assess and address systemic issues which may have a more direct impact on stress and burnout. This focus should have an additive effect on the SFA goal of increasing awareness of stress injuries, helpful self-care, and peer support actions, resources, and access to care, as well as its goal of helping to identify, assist, and link into care those individuals who may be at risk for developing stress injuries that could predispose them to longer-term mental health conditions. Future studies should inspect the longer-term impact of SFA utilization across a healthcare system and potential company-wide cultural shifts in the discussion of stress and burnout.

### 4.2. Practical Implications

The findings from this study are significant because, in contrast to prior studies [33,35], this study protocol committed to implementing SFA in the recommended flexible way, with local SFA Coaches supported by more experienced supervisors. This approach yielded greater self-efficacy, resilience, and resource knowledge than studies with a more controlled, rigid format with less supervision [33,35]. This makes the current findings and guidelines more generalizable to the recommended implementation of SFA in high-stress work environments. Organizations that have the time and dedicated resources to follow up with multiple training opportunities, support from SFA Coaches, and multi-tiered ways to implement SFA actions should find better results in reduction in stress, increased coping self-efficacy, and perceived social and organizational support, mainly when initiated before public health emergencies rather than during a pandemic.

As noted previously, SFA is in alignment with implementation principles recommended for complex environments and public health emergencies, such as using research- or theory-informed frameworks to empower adaptive actions within a flexible approach to solve problems that a system cannot yet anticipate [47]. Building upon existing informal networks of support with a built-in level of trust allows for faster implementation, and greater trust allows HCWs to adapt actions to their moment-to-moment capacity. SFA, implemented this way, allows individuals and systems to define and move towards reduced stigma and enhanced communication about the ongoing necessary actions toward greater wellbeing and system effectiveness.

### 4.3. Limitations and Future Directions

The results of this study should be interpreted considering several limitations, with implications for future research. First, the present study had a relatively small sample size. Power was sufficient for the proposed statistical analyses; increasing the sample size in future studies may allow for group differentiation such as by role type, gender, and other variables. Additionally, a larger sample size or oversampling for additional demographic variables or variables of otherwise interest would have allowed for a more nuanced understanding of the effects of SFA among healthcare workers. The current study focused on healthcare workers in a behavioral health setting without specific control or comparison groups. For example, comparing study findings to control groups of other healthcare workers outside of a psychiatric setting may have been advantageous. While our outcomes assessment followed the proximal and distal outcomes of the Stress First Aid intervention, future repeated assessment of variables would strengthen causal inference and potential temporal mediation models. Whereas the present study focused on several proximal outcomes associated with occupational stress and burnout, future research might investigate additional outcomes. Research has found associations between occupational stress and other behavioral health outcomes, including substance use disorders and depression [54] among healthcare workers.

## 5. Conclusions

This study sought to extend the use of the peer support intervention, Stress First Aid, to healthcare workers during the COVID-19 pandemic. Findings provide preliminary support for the potential efficacy of SFA as a means of shifting culture around the discussion of emotional wellbeing, as well as an awareness and utilization of employee health resources. It will be important to continue to observe the potential impact of SFA during non-pandemic times as it has been used as a resilience-building, prevention strategy in military and first responder settings. It is possible that in less critical times, the impact on the more distal outcomes would be easier to observe because there would be fewer competing stressors. Further, results support the need to investigate implementation, dissemination and sustainability indicators as those will be integral in wider adoption of the model.

## Figures and Tables

**Table 1:** Occupational role breakdown of unique respondents.

	Frequency	Percent
RN—Registered Nurse	96	36.1
Mental Health Nursing Staff	90	33.8
Unit Receptionist	20	7.5
ANM—Assistant Nurse Manager	14	5.3
Other	12	4.5
Nursing Administrator	9	3.4
NM—Nurse Manager	9	3.4
Scheduler	4	1.5
PCA—Patient Care Associate	3	1.1
Director	3	1.1
Nursing Attendant	2	0.8
Nurse Educator	2	0.8
NA—Nursing Assistant	1	0.4
Certified Nursing Assistant	1	0.4
Total	266	100

**Table 2:** Outcome measures based on the least square means across all time Points.

	Baseline		3-Month Follow-Up		6-Month Follow-Up		9-Month Follow-Up		12-Month Follow-Up	
	M	SE	M	SE	M	SE	M	SE	M	SE
SFA Self-Efficacy *	9.10	0.15	9.15	0.13	9.36	0.14	9.21	0.14	9.54	0.14
Resilience **	8.28	0.11	8.13	0.12	8.13	0.13	8.17	0.12	8.47	0.11
Perceptions of Organizational Support	7.89	0.16	7.94	0.15	7.99	0.15	8.16	0.16	8.29	0.14
Stress	2.18	0.07	2.11	0.06	2.07	0.07	2.14	0.07	2.10	0.07
Burnout	5.11	0.13	4.99	0.14	5.20	0.17	5.85	0.15	4.82	0.14
Resource Awareness **	4.32	0.26	4.61	0.25	4.98	0.28	4.99	0.28	5.28	0.29
Any Resource Utilization (OR; 95% CI)	0.27	(0.18–0.40)	0.28	(0.19–0.43)	0.31	(0.20–0.48)	0.33	(0.21–0.53)	0.40	(0.27–0.60)

Note: * *p* = 0.05, ** *p* < 0.01.

## Data Availability

The data presented in this study are available by request and sent to the corresponding author.
